# A cross-sectional survey exploring HIV and HCV prevalence among men who purchase sex in Dnipro, Ukraine

**DOI:** 10.1186/s12889-023-16903-1

**Published:** 2023-10-20

**Authors:** Lisa Lazarus, Nicole Herpai, Daria Pavlova, Amaanat Gill, François Cholette, Leigh M. McClarty, Shajy Isac, Anna Lopatenko, Michael Pickles, Sharmistha Mishra, Souradet Y. Shaw, Robert Lorway, Lyle R. McKinnon, Paul Sandstrom, James Blanchard, Olga Balakireva, Marissa L. Becker, on behalf of the Dynamics Study Team, Sevgi Aral, Tetiana Bondar, Eve Cheuk, Christina Daniuk, Evelyn Forget, Emma Lee, Huiting Ma, Stephen Moses, Maureen Murney, Nam-Mykhailo Nguien, Ani Shakarishvili, Tatiana Tarasova

**Affiliations:** 1 Institute for Global Public Health, Rady Faculty of Health Sciences, University of Manitoba, R070 Med Rehab Building, 771 McDermot Ave., Winnipeg, Manitoba R2E 0T6 Canada; 2 Department of Community Health Sciences, University of Manitoba, Winnipeg, Canada; 3 Ukrainian Institute for Social Research after Oleksandr Yaremenko, Kyiv, Ukraine; 4 Department of Medical Microbiology and Infectious Diseases, University of Manitoba, Winnipeg, Canada; 5 Sexually Transmitted and Blood-Borne Infections, National Microbiology Laboratory at JC Wilt Infectious Diseases Research Centre, Public Health Agency of Canada, Winnipeg, Canada; 6 India Health Action Trust, Delhi, India; 7 Dnipropetrovsk Regional Center for Socially Significant Diseases, Dnipro, Ukraine; 8 Department of Infectious Disease Epidemiology, School of Public Health, Imperial College London, London, UK; 9 MAP Centre for Urban Health Solutions, Li Ka Shing Knowledge Institute, St. Michael’s Hospital, Toronto, Canada; 10 Department of Medicine, University of Toronto, Toronto, Canada; 11 Institute of Medical Sciences, University of Toronto, Toronto, Canada; 12 Institute of Health Policy, Management and Evaluation, University of Toronto, Toronto, Canada; 13 Centre for the AIDS Programme of Research in South Africa (CAPRISA), Durban, South Africa; 14 Department of Medical Microbiology & Immunology, University of Nairobi, Nairobi, Kenya; 15 Institute for Economics and Forecasting, Ukrainian National Academy of Sciences, Kyiv, Ukraine

**Keywords:** Sex work, Men who purchase sex, Clients, HIV, HCV, Ukraine

## Abstract

**Background:**

HIV programming in Ukraine largely targets “key population” groups. Men who purchase sex are not directly reached. The aim of our study was to explore the prevalence of sexually transmitted and blood-borne infections (STBBIs) among men who purchase sex from female sex workers.

**Methods:**

Following geographic mapping and population size estimation at each “hotspot”, we conducted a cross-sectional bio-behavioural survey with men who purchase sex between September 2017 and March 2018 in Dnipro, Ukraine. Eligibility criteria included purchasing sex services at a “hotspot” and being ≥ 18 years. Participants completed a structured questionnaire, followed by HIV/HCV rapid testing and a dried blood spot (DBS) sample collection for confirmatory serology.

**Results:**

The study enrolled 370 participants. The median age was 32 (interquartile range [IQR] = 27–38) and the median age of first purchase of sexual services was 22 (IQR = 19–27). Over half (56%) of participants reported ever testing for HIV; four participants (2%, *N* = 206) reported having tested positive for HIV, with three out of the four reporting being on ART. Forty percent of participants had ever tested for HCV, with three (2%, *N* = 142) having ever tested positive for HCV. In DBS testing, nine participants (2.4%) tested positive for HIV and 24 (6.5%) tested positive for ever having an HCV infection.

**Conclusion:**

Prevalence of HIV and HCV in this population was high. Given high rates of study enrolment and testing, efforts should be made to reach men who purchase sex with expanded STBBI programming.

## Background

HIV prevention programming in Ukraine has historically mostly been funded by external donors such as the Global Fund to Fight AIDS, Tuberculosis and Malaria, and implemented by non-governmental organizations (NGOs) such as the Alliance for Public Health and the All-Ukrainian Network of People Living with HIV/AIDS (now named 100% Life) [1, 2]. These programs target “key population” groups, including people who inject drugs, men who have sex with men, and female sex workers [1, 3, 4]. The responsibility for funding HIV programming began to gradually transition to the Government of Ukraine in 2018, adding to the role the government already played in delivering HIV treatment [1]. However, Ukraine continues to fall short of global HIV “test and treat” targets [1]. UNAIDS has reported that people in Ukraine are often diagnosed at a late stage of infection; 6.6% of the population had an HIV test in 2019, with 48% of these individuals being either pregnant women or blood donors [5]. Men who purchase sex from female sex workers are generally not directly reached by these programs.

Interventions designed to reach men who purchase sex are still rare in most settings globally [6–8]. Research on clients of female sex workers largely focuses on HIV and other sexually transmitted and blood borne infection (STBBI) prevalence, condom use, number of sex partners, and drug use [6–13]. Qualitative studies from Indonesia have highlighted barriers to HIV testing, such as fears surrounding stigma and discrimination, gender norms around masculinity and strength, a lack of knowledge surrounding HIV, denial about HIV vulnerability, and a lack of availability of HIV treatment, despite the availability and free cost of testing [14, 15]. In exploring HIV testing and treatment in Sub-Saharan Africa, Shand and colleagues have called men “the HIV blind spot”, highlighting the role that gender inequality plays in HIV transmission, while noting that a focus on gender has often translated into policies, programs, and funding focused squarely on women’s health [16]. The authors underscore the “limited attention” that has been dedicated towards addressing men’s needs and health-seeking behaviours related to HIV prevention, testing, and treatment, noting that:

There is, nevertheless, increased understanding that unhealthy constructions of masculinity – male gender norms associated with toughness and control, sexual prowess and heteronormativity, as a way of asserting manhood – can deter men from seeking HIV care and support, even at times of vulnerability and ill health… [16] (p.53).

Limited data are available on men who purchase sex in Ukraine. A survey from 2009 estimated an overall HIV prevalence of 7.4% among clients of female sex workers; 23% among those reporting injection drug use and 3% among those stating that they did not use injection drugs [17]. This is significantly higher than the estimated prevalence of approximately 1% among the adult population (men and women, aged 15–49 years) [18]. Since 2014, there has been a continuing state of conflict in the Donbas region in eastern Ukraine, leading to concerns that the conflict may pose worsening challenges in addressing HIV and STBBIs due to cuts in health and social programs [19]. The aim of our study was to explore the prevalence of STBBIs, including HIV and hepatitis C virus (HCV), among men who purchase sex in Dnipro, Ukraine.

## Methods

### Study setting

The Dynamics study is a cross-sectional mixed methods study exploring the impact of conflict on HIV and HCV risk and prevalence among female sex workers and men who purchase sex in Dnipro city, Dnipropetrovsk oblast (study details have been described in detail in a previous publication [20]). Dnipro has a population of nearly 1 million people, with 45.2% of the population being male [21]. There are an estimated 86,600 sex workers working across Ukraine [18]. HIV cases in Dnipropetrovsk region are estimated at 765.0 per 100,000 population, compared to the national average of 355.1 per 100,000 population [5].

### Data collection

The Dynamics study is a partnership between the NGO “Ukrainian Institute for Social Research after Oleksandr Yaremenko” (UISR after Oleksandr Yaremenko) and the Institute for Global Public Health at the University of Manitoba, with collaboration from the Center for Public Health of the Ministry of Health in Ukraine and the Dnipro Oblast AIDS Center. Local data collection was undertaken by researchers from UISR after Oleksandr Yaremenko and DEF Group (a research organization-based in Dnipro).

Geographic mapping and population size estimation was conducted in “hotspots” that are associated with sex work (details on mapping are described in previous publications [22, 23]; details on hotspots are described in another publication [24]). Mapping and size estimation involved a two-stage approach. In the first stage, a list of potential hotspots was generated through interviewing key informants who are associated with, or aware of, workplaces for sex work. Key informants included sex workers, other individuals connected to sex work, and people working in NGOs. In the second stage, each identified hotspot was visited by the mapping team (consisting of a researcher and a social mobilizer, usually an outreach worker) to validate its existence and activity, and estimate population size. The mapping exercise captured hotspot types and estimates of the number of women working at each hotspot, and the number of men who were at the hotspot to purchase sex services. This list of 320 active hotspots formed the basis for the sampling frame for the bio-behavioural assessment. The mapping exercise estimated 1,395 female sex workers (range 1,155–1,635) and a mean of 9.2 men who intended on buying sex services (interquartile range [IQR] 5.0–11.0) per hotspot. Following mapping, we conducted a cross-sectional bio-behavioural survey between September 2017 and March 2018. Men were selected to participate following a two-stage study design, selecting hotspots in the first stage and participants in the second stage. The sample of hotspots were selected using a probability proportion to size of estimated men at the hotspot and then subsequently, men were selected randomly from the sampled hotspots. Study interviewers, with the help of social mobilizers, recruited participants. Inclusion criteria for men was purchasing sex at the validated hotspot and being ≥ 18 years. Following written informed consent for eligible participants, trained interviewers administered a structured questionnaire in the local language. Survey questions included standardized and locally validated questions on socio-demographics, risk behaviours, and structural factors based on Integrated Biological and Behavioural Survey Guidelines, and our prior work [25, 26]. Surveys took, on average, thirty minutes to complete and participants received an honorarium of 400 UAH (approximately $20 CDN) for their time.

For consenting participants, biological tests included a fingerprick sample to be used in rapid tests for HIV (SD Bioline HIV ½ 3.0) and HCV (Wantai Rapid test for HCV), as per national testing guidelines. Pre- and post-test counselling was provided, as well as linkage to treatment and care services for those who tested positive for HIV and HCV. A dried blood spot (DBS) sample was also collected for confirmatory HIV and HCV serological testing (Avioq HIV-1 Microelisa System and Ortho HCV v3.0 ELISA Test System respectively) and viral genotyping [27, 28] at the National HIV and Retrovirology Laboratories in Winnipeg, Canada. Prevalence results were calculated based on confirmatory serological testing.

### Data analysis

Descriptive statistics and measures of central tendency were performed using Stata15.

### Ethics

Written informed consent was obtained for all participants. Ethical approval was obtained from the Human Research Ethics Board at the University of Manitoba [HS20653(H2017:097)], the Ethical Review Committee of the Sociological Association of Ukraine, and the Committee of Medical Ethics of the L. Gromashevsky Institute of Epidemiology and Infectious Diseases at the National Academy of Medical Sciences of Ukraine.

## Results

Based on our sampling frame, the study team approached 416 men to participate in the study. Of those, 46 declined to participate and 370 (88.9%) were enrolled in the study from 117 different spots, including a range of venue types (from highways to indoor establishments). Participant characteristics, sex and drug use practices, and HIV and HCV testing and prevalence are detailed in the following sections.

### Participant characteristics

Participant characteristics are summarized in Table 1. The median age of study participants was 32 years (interquartile range [IQR] = 27–38 years). Over half of participants were single, widowed, divorced, or not living with an intimate partner. Approximately a third of participants (32.2%, *n* = 119) had completed graduate school and a third were entrepreneurs (33.2%, *n* = 123). Almost half of the men had enough money to buy necessities such as food, clothing and shoes, and other basic items, but had to save, borrow money, or take a loan to purchase expensive items (e.g., large household appliances—TV, refrigerator, computer, washing machine, etc.) (47.6%, *n* = 176).

**Table 1:** Participant characteristics

Age in years (*N* = 370)	n (%)
< 20	16 (4.3)
20–24	47 (12.7)
25–29	87 (23.5)
30–34	72 (19.5)
35–39	71 (19.2)
≥ 40	77 (20.9)
Marital status (*N* = 370)	
Single	133 (36.0)
Married and living with spouse	90 (24.3)
Married and not living with spouse	38 (10.3)
Not married but living with an intimate partner	32 (8.7)
Widowed or divorced	73 (19.7)
Occupation (*N* = 370)	
Civil servant	7 (1.9)
Top-level manager	6 (1.6)
Technical specialist with higher education^a^	60 (16.2)
Professional job^b^	16 (4.3)
Law enforcement, military officer	19 (5.1)
A self-employed entrepreneur of a small, medium, or large business	123 (33.2)
General labourer	71 (19.2)
Hospitality (bartender, cook), sales	9 (2.4)
Truck/long haul driver, taxi driver	11 (3.0)
Student	28 (7.6)
Unstable employment, unemployed, pensioner	12 (3.2)
Other	8 (2.2)
Education (*N* = 370)	
Incomplete secondary education (9 grades)	2 (0.5)
Completed secondary education (11 grades)	14 (3.8)
Vocational training with completed secondary education	50 (13.5)
Specialized technical education with a diploma of a junior specialist (technical school, college, etc.)	114 (30.8)
Bachelor’s degree or incomplete graduate school	71 (19.2)
Completed graduate school (Masters, PhD)	119 (32.2)
Financial situation (*N* = 370)	
I have enough money to buy food, but I have to save or borrow money to buy other necessities such as clothing and shoes	15 (4.1)
I have enough money to buy necessities such as food, clothing, and shoes, but I have to save, borrow money, or take a loan to purchase basic items such as a mobile phone, small household appliances (e.g., iron, vacuum cleaner, etc.)	154 (41.6)
I have enough money to buy necessities such as food, clothing and shoes, and other basic items, but I have to save, borrow money, or take a loan to purchase expensive items (e.g., large household appliances—TV, refrigerator, computer, washing machine, etc.)	176 (47.6)
I have enough money to buy necessities such as food, clothing and shoes, and other expensive items but I have to save, borrow money, or take a loan to purchase very expensive items (e.g., a new car, holiday home, etc.)	21 (5.7)

^a^ for example: engineer, mechanic, electrician, IT specialist

^b^ for example: doctor, teacher, professor

### Sex and drug use practices

The median age of first purchase of sexual services was 22 years (IQR = 19–27 years). Almost half of the men visited “offices”, a brothel-like venue, as their main place to purchase sex services (48.9%, *n* = 181); slightly more than half visited only one hotspot (55.1%, *n* = 204) and 44.1% (*n* = 163) visited more than one hotspot in the past 12 months. Two-thirds of men (66.2%, *n* = 245) reported seeing only one sex worker in the past 30 days. Condom use was high, with 95.9% (*n* = 347) of participants reporting using a condom 100% of the time with sex workers in the past 30 days, while 77.5% (*n* = 287) reported never paying extra for sex without a condom in the past 12 months. Most men had sex with other partners in the previous 30 days, including intimate, transactional, and/or casual sex partners (84.9%, *n* = 314). Of those who had sex with intimate partners, 39.7% (*n* = 106) did not use condoms.

Over a third of participants (38.1%, *n* = 141) had ever used illicit drugs; 14 (9.9%) of whom ever used injection drugs. Half of the participants “binge-drank” (defined as consumed 5 or more alcoholic drinks in a row) at least once a month (49.7%, *n* = 184) (Table 2).

**Table 2:** Sex and drug use practices

Age in years at first purchase of sex services (*N* = 370)	n (%)
< 20	129 (34.9)
20–24	108 (29.2)
25–29	72 (19.5)
30–34	28 (7.6)
35–39	20 (5.4)
≥ 40	13 (3.5)
Main hotspot type visited to purchase sex services in past 12 months (*N* = 370)	
"Office" (brothel)	181 (48.9)
Apartment	48 (13.0)
Cafe, restaurant, bar	41 (11.1)
Highway	27 (7.3)
Street	20 (5.4)
Massage, salon, sauna	19 (5.1)
Art-club, strip club	13 (3.5)
Venue	10 (2.7)
Hotel/motel	6 (1.6)
Other	5 (1.4)
Number of hotspots visited to purchase sex services in the past 12 months (*N* = 370)	
1	204 (55.1)
2	41 (11.1)
3	58 (15.7)
4	37 (10.0)
≥ 5	27 (7.3)
Number of times sex services were purchased from a sex worker in the past 30 days (N = 370)	
0	7 (1.9)
1	224 (60.5)
2	96 (26.0)
≥ 3	42 (11.4)
Number of different sex workers that participants had sex with in the past 30 days (*N* = 370)	
0	8 (2.2)
1	245 (66.2)
2	89 (24.1)
3	21 (5.7)
≥ 4	7 (1.9)
Consistent condom use when with a sex worker in the past 30 days (*N* = 362)	347 (95.9)
Paid extra for sex without a condom with sex workers in the past 12 months (*N* = 370)	
All the time/Most of the time	33 (8.9)
Half the time/Sometimes	24 (6.5)
Rarely	21 (5.7)
Never	287 (77.5)
Number of other sex partners (intimate, casual and transactional^a^) in the past 30 days (*N* = 370)	
0	56 (15.1)
1	183 (49.5)
2	69 (18.7)
3	38 (10.3)
≥ 4	24 (6.5)
Participants who did not use a condom with intimate partners in the past 30 days (*N* = 267)	106 (39.7)
Frequency of binge-drinking^b^ in the past 30 days (*N* = 370)	
At least once a week	34 (9.2)
1 to 3 times a month	150 (40.5)
Other	10 (2.7)
I have never binge-drank	138 (37.3)
I quit drinking/I did not drink alcohol	35 (9.5)
Ever used illicit drugs (*N* = 370)	141 (38.1)
Ever injected drugs (*N* = 141)	14 (9.9)
Last time drugs were injected (*N* = 14)	
Within last 12 month	5 (35.7)
More than one year ago	8 (57.1)

^a^ Transactional sex partners refer to women that men had sex with where there was an expectation that money, gifts, or other resources would be provided but no set price of sex was negotiated beforehand

^b^ Binge-drinking is defined as consuming 5 or more alcoholic drinks in a row

### STBBI testing and treatment

Among participants, over half (56.2%, *n* = 208) reported ever testing for HIV in their lifetime, nearly all of whom (99.0%, *n* = 206) had received the result from their last HIV test. Two percent (*n* = 4) reported having tested positive for HIV. Three out of the four participants who had previously tested positive for HIV had started antiretroviral therapy (ART) and were on ART at the time of the survey. Among those who had ever tested, approximately a quarter (26.4%, *n* = 55) had tested within the past year. Forty percent (*n* = 148) of participants had ever tested for HCV; 95.9% (*n* = 142) had received results from their last HCV test, with two percent (2.1%, *n* = 3) having ever tested positive for HCV. Sixteen percent (15.7%, *n* = 58) of participants self-reported ever having a sexually transmitted infection (STI), such as chlamydia, gonorrhea, or syphilis. In the past 12 months, three (0.8%) participants reported being treated for an STI.

### Rapid and dried blood spot testing for HIV and HCV

All participants (100%, *n* = 370) provided consent to receive a rapid test for HIV and HCV, and all but one (99.7%, *n* = 369) agreed to a DBS test for HIV and HCV testing. Nine participants (2.4%) tested positive for HIV in both the rapid and DBS test; 32 (8.7%) tested positive for HCV using the rapid test and 24 (6.5%) tested positive for HCV in the DBS. It is important to note that a positive HCV test result indicates having ever tested positive for HCV and is not necessarily indicative of an active infection.

Of the nine participants who tested positive for HIV, five did not self-report a previous positive HIV test result. Among the 24 participants who tested positive for HCV, 23 (95.8%) did not report a previous positive HCV test result. In sub-analyses, two of those testing positive for HIV and six of those testing positive for HCV reported ever using injection drugs.

## Discussion

In our study, men who purchased sex principally visited “offices” and for the most part frequented only one venue. Reported condom use with sex workers was high and few men paid extra for sex without a condom. Most participants had additional sex partners such as intimate, transactional, and/or casual sex partners. Over half of the participants had tested for HIV at least once in their lifetime, while less than half had ever tested for HCV. Of the four participants who had previously tested positive for HIV, three were on ART. In this study, nine participants tested positive for HIV and 24 tested positive for ever having an HCV infection.

Sex worker interventions, including examples from India and Thailand, have been successful in reaching clients with STBBI prevention through collaborative efforts with sex workers and sex business owners [29–33]. However, generally, men who purchase sex are rarely directly included in STBBI prevention and care programming, unless they also identify as a “key population” group, such as people who use drugs. Concerted efforts need to be made to reach men with STBBI prevention, testing, and treatment services. While barriers to accessing HIV prevention and care for men who purchase sex have been noted [15, 16, 34], Patterson et al. [7] have highlighted the urgent need for interventions that reach men who purchase sex with STBBI programming that also addresses masculinity and drug use, and socio-structural and individual-level risks, to meet different cultural contexts and needs (also discussed by Pitpitan and colleagues [35]). Similarly, Shand and colleagues note that:

In understanding masculinities and HIV, it is critical to situate the HIV epidemic not just within gender-inequitable dynamics at an individual level, but also within the larger male-centric power structures that inequitably drive the epidemic [36] and the all-encompassing expectation of male authority that limits men’s ability to show vulnerability [16] (p.53)

While men may often benefit from economic and political advantages over women, they are less likely to engage in HIV-related care and more likely to face HIV-related mortality [16]. Our study had a significantly high response rate of 88.9%. Additionally, all participants consented to rapid testing and only one declined DBS testing for HIV and HCV. Despite reports of low HIV testing rates among the general population in Ukraine [1, 5], our study demonstrates a potential for reaching men who purchase sex with expanded STBBI prevention and programming, including testing—a necessary first step in meeting global test and treat targets. In Malawi, engagement with men who purchase sex in order to understand and tailor services to them showed that men had a variety of preferences on where and when to access HIV services, demonstrating the importance of user engagement early in program planning [33].

Dramatic societal changes in post-conflict settings can increase vulnerability to HIV and other STBBIs as population movement occurs and social networks reintegrate [37]. While a transition to government services followed by ongoing structural shocks such as the COVID-19 pandemic and full-scale war with Russia pose very real challenges to HIV programming in Ukraine [2], once peacetime resumes it will be critical to engage men in STBBI prevention, testing, and care. Furthermore, sex work is criminalized or quasi-criminalized in most global settings, including Ukraine, with the laws and policies surrounding sex work creating harm for sex workers [38, 39]. These criminalized contexts extend to targeting men who purchase sex, creating significant barriers for accessing prevention, testing, and care [40–42]. Continuing country-level discussions on the decriminalization of sex work [43, 44] and ensuring that decriminalization of sex work includes people who purchase sex services and others working in the sex industry, is necessary to decrease barriers to accessing HIV prevention and treatment among men.

### Limitations

These data are descriptive in nature and drawn from a cross-sectional bio-behavioural survey, therefore no causality can be inferred. Participant characteristics, sex and drug use practices, and previous STBBI testing and treatment are self-reported and subject to recall, social desirability, and misclassification biases. Since the list of hotspots identified in the mapping served as the sampling frame for this study, the men recruited from mapped hotspots and who agreed to participate in the study may not represent men who purchase sex outside of these hotspots or who chose not to participate. Further, these data were collected between 2017 and 2018 and do not reflect the current situation in Dnipro, Ukraine, which is undergoing a severe crisis due to full-scale, nation-wide war.

## Conclusion

Prevalence of HIV and HCV in this population was higher compared to the general population, despite participants reporting consistent condom use and high levels of testing when compared to the general population. Given high rates of study enrolment and testing, efforts should be made to reach men who purchase sex with expanded STBBI programming. This study gives insights into the potential to reach men with a range of services, including STBBI prevention and treatment.

## Data Availability

The datasets used and/or analysed during the current study are available from the corresponding author on reasonable request.
